# Self-Assembly of Lamellar/Micellar Block Copolymers Induced Through Their Rich Exposure to Various Solvent Vapors: An AFM Study

**DOI:** 10.3390/ma18081759

**Published:** 2025-04-11

**Authors:** Iulia Babutan, Leonard Ionut Atanase, Ioan Botiz

**Affiliations:** 1 Interdisciplinary Research Institute on Bio-Nano-Sciences, Babeș-Bolyai University, 400271 Cluj-Napoca, Romania; iulia.babutan@ubbcluj.ro; 2 Department of Physics of Condensed Matter and Advanced Technologies, Faculty of Physics, Babeș-Bolyai University, 400084 Cluj-Napoca, Romania; 3 Department of Biomaterials, Faculty of Medicine, “Apollonia” University of Iasi, 700511 Iasi, Romania; leonard.atanase@univapollonia.ro; 4 Academy of Romanian Scientists, 050045 Bucharest, Romania

**Keywords:** block copolymers, thin films, solvent vapor annealing, hierarchical self-assembly, atomic force microscopy

## Abstract

In this work, we have employed an advanced method of solvent vapor annealing to expose spin-cast thin films made from various lamellar and micellar block copolymers to generous amounts of different types of solvent vapors, with the final goal of stimulating the films’ self-assembly into (hierarchically) ordered structures. As revealed by atomic force microscopy measurements, periodic lamellar nanostructures of molecular dimensions based on poly(4-vinylpyridine)-*b*-polybutadiene and poly(2-vinylpyridine)-*b*-polybutadiene, as well as micellar structures further packed into either (parallel) stripe-like or honeycomb-resembling configurations based on poly(2-vinylpyridine)-*b*-poly(tert-butyl methacrylate)-*b*-poly(methacrylate cyclohexyl), were successfully produced through processing.

## 1. Introduction

Block copolymers (BCPs) are macromolecules composed of two or more chemically distinct homopolymer segments that are covalently linked together, while each segment may contain dozens to hundreds of (identical) repeating units. These blocks can be arranged in various topologies, leading to highly diverse molecular architectures, including branched, cyclic, and hybrid structures [1,2,3,4]. Moreover, BCPs have the ability to self-assemble into unique macromolecular structures such as lamellae, spheres/particles, cylinders, or gyroids [1], just to name a few. Self-assembly is more efficient for BCPs displaying precisely controlled compositions and molecular weights and typically proceeds through the thermodynamic phase separation of their incompatible segments [5,6]. In particular, nanostructures resulting from microphase separation in thin films of BCPs have gained significant interest as templates and masks for the fabrication of periodic (hierarchical) nanostructures in functional materials [7,8,9,10].

One of the primary advantages of BCPs is the possibility to control the orientation of the resulting microphase-separated nanostructures using various external stimuli, such as light, substrate topological patterns, or interfacial energy, which favors the self-assembly of long macromolecules into highly ordered structures [11,12,13]. Such structures hold great promise in various advanced practical applications, including magnetic data storage and optoelectronic [14,15,16], biomedical/health-related [17,18,19,20], and other [21,22] types of devices. To continuously diversify BCP-based applications, researchers have actively pursued innovative and efficient strategies to generate nanostructures with precise control. The process of self-assembly of BCPs represents a powerful method that can lead, at reduced costs, to highly ordered periodical nanostructures as small as a few nanometers [23,24] (note that some defects, such as disclinations, dislocations, and point defects at grain boundaries, might still be unavoidable on large area samples [25,26,27,28,29]) that, for instance, further display a tremendous potential in the current patterning technology [30].

Some of the most common BCP-based structures include lamellar [31] and micellar [32,33,34,35] morphologies obtained through the utilization of good or selective solvents, and these are mainly favored when utilizing the appropriate processing methods such as solvent vapor annealing (SVA). SVA is one of the most prominent tools [36] for achieving long-range ordered structures in thin film BCP systems [37,38,39,40,41,42], including spheres or lamellae [43], and is especially valuable for BCPs displaying a high Flory–Huggins interaction parameter (*χ*) when targeting the generation of microdomain patterns with small periodicity [37,38,39,44,45]. For instance, the resulting morphology in thin BCP films is primarily influenced by factors such as *χ*, the degree of polymerization (*N*), the volume fraction (*f*) [46,47,48], or surface/confinement effects [49]. However, SVA introduces additional tunability of morphology by controlling the type of solvent and time of exposure to solvent vapors, as well as the composition of solvent mixtures and their selectivity toward different polymer blocks [41,50,51].

In this work, we employ an advanced processing technique known as space-confined solvent vapor annealing (C-SVA) [52,53,54] to self-assemble, into lamellar and micellar morphologies, a variety of diblock and triblock copolymers, based on polybutadiene, poly(4-vinylpyridine), poly(2-vinylpyridine), poly(tert-butyl methacrylate, poly(methacrylate cyclohexyl), or poly(acrylic acid) blocks, by exposing thin films of the corresponding BCPs to a rich amount of solvent vapors, followed by regulation of the rate of solvent evaporation. The processing is conducted in a custom-built setup comprised of a shallow sample chamber filled with solvent vapors and housing both the BCP film and a temperature sensor. The latter, along with a highly precise temperature controller, enables us to achieve and maintain any film temperature between −15 °C and 65 °C, with a control accuracy of ±0.01 °C. This setup, well described elsewhere [52,53,54], allows solvent vapors to be precisely condensed on the film surface and thus, any BCP film to swell until it becomes a quasi-2D “film solution”. Exerting control over the film deswelling process, i.e., specifically the rate of vapor extraction, induces and favors the self-assembly process that leads to highly ordered lamellar or micellar BCP morphologies. For example, micellar systems are of significant interest in biomedical applications [33,55,56,57,58,59]. While most studies focus on aqueous micellar systems, research into non-aqueous micellar systems is equally important due to its potential for large-scale applications in membrane technology, pigment surface modification, non-aqueous dispersions, etc. Moreover, SVA, along with other processing methods, is frequently utilized to self-assemble various BCPs on surfaces via microphase-separation, with the goal of generating not only (cylindrical, wormlike, spherical) micellar structures but also vesicles [60] and lamellar morphologies that can be applied in various pattern transfer technologies [61,62,63,64,65,66].

## 2. Materials and Methods

**Figure 1:**
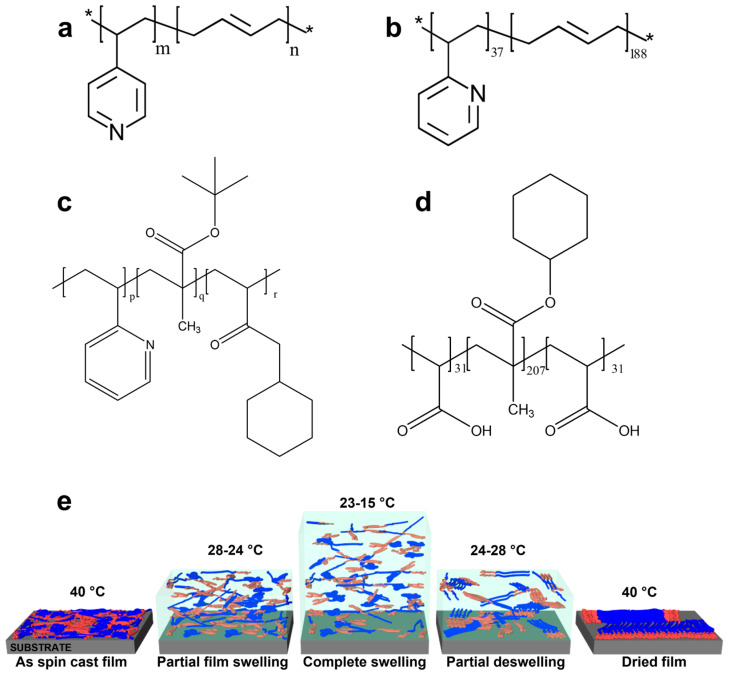
Chemical structures of P4VP-*b*-PB (**a**), P2VP-*b*-PB (**b**), P2VP-*b*-P*t*BMA-*b*-PCHMA (**c**), and PAA-*b*-PCHMA-*b*-PAA (**d**) BCPs studied in this work. Here, indices represent the number of corresponding repeating monomer units (*m* is 34 or 43, *n* takes the values of 70 or 207, *p* represents 25 or 107, *q* is 12 or 52, *r* replaces 173 or 604). (**e**) Schematics illustrating the expected mechanism of self-assembly during the film swelling/deswelling procedure conducted through C-SVA.

Poly(4-vinylpyridine)-*b*-polybutadiene (P4VP-*b*-PB), poly(2-vinylpyridine)-*b*-polybutadiene (P2VP-*b*-PB), poly(2-vinylpyridine)-*b*-poly(tert-butyl methacrylate)-*b*-poly(methacrylate cyclohexyl) (P2VP-*b*-P*t*BMA-*b*-PCHMA), and poly(acrylic acid)-*b*-poly(cyclohexyl methacrylate)-*b*-poly(acrylic acid) (PAA-*b*-PCHMA-*b*-PAA) are di-BCPs and tri-BCPs utilized in this work (see their chemical structures illustrated in Figure 1a–d). Their average molecular weight (*M_n_*) is as follows: 14,800 g/mol for P4VP_34_-*b*-PB_207_, 8400 g/mol for P4VP_43_-*b*-PB_70_, 14,100 g/mol for P2VP_37_-*b*-PB_188_, 120,000 g/mol for P2VP_107_-*b*-P*t*BMA_52_-*b*-PCHMA_604_, 33,300 g/mol for P2VP_25_-*b*-P*t*BMA_12_-*b*-PCHMA_173_, and 39,200 g/mol for PAA_31_-*b*-PCHMA_207_-*b*-PAA_31_. All BCP systems were synthesized through anionic polymerization in tetrahydrofuran (THF) in the presence of n-Butyllithium (n-BuLi) at −75 °C, following well-established procedures detailed in previous studies [35,67,68].

To prepare the copolymer solutions, toluene (C_6_H_5_CH_3_, 98%), 1,4-dioxane (C_4_H_8_O_2_, 99.5%), 1,2-dichloroethane (1,2-DCE, C_2_H_2_Cl_2_, 97%), chloroform (CHCl_3_), acetone (C_3_H_6_O, 99.5%), tetrahydrofuran [THF, (CH_2_)_4_O, 99.8%], and methanol (CH_3_OH) reagents (purchased from Chemical Company Iasi, Romania) were used. THF and methanol were also used in the form of a mixture, in a molar volume of 1:1. Each copolymer solution was prepared by dissolving 10 mg of copolymer powder in 1 mL of solvent, followed by gentle stirring of the resulting mixture. To ensure complete dissolution of the copolymer powder in the solvents, the polymer solutions were often heated to 70 °C in a silicone oil bath (ONE 7-45, Schwabach, Germany) for 30 min. Furthermore, thin BCP films of varying thicknesses were fabricated by casting each of the obtained copolymer solutions onto silicon wafers (type P/B-doped, with resistivity ranging from 5 to 10 Ω·cm and a thickness of 380 ± 15 µm, from Siegert Wafer GmbH, Aachen, Germany) and using a spin coater from Laurell Technologies Corporation (model WS-650mz23nppb, North Wales, PA, USA) running at a speed of 2000 rpm for 30 s (a typical acceleration of 500 rpm/s was employed). Thin BCP films of a thickness of ~97 nm (P2VP_37_-*b*-PB_188_), ~105 nm (P4VP_34_-*b*-PB_207_), ~95 nm (P4VP_43_-*b*-PB_70_), ~99 nm (P2VP_25_-*b*-P*t*BMA_12_-*b*-PCHMA_173_), ~106 nm (P2VP_107_-*b*-P*t*BMA_52_-*b*-PCHMA_604_), and ~112 nm (PAA_31_-*b*-PCHMA_207_-*b*-PAA_31_) were obtained. All film thicknesses were determined using atomic force microscopy (AFM) by measuring the depth profiles of scratches intentionally generated on the film surfaces. Prior to use, all silicon wafers underwent UV ozone treatment (performed utilizing PSD Pro Series-Digital UV Ozone System from Novascan, Boone, IA, USA) for 20 min to ensure they were cleaned and hydrophilized.

Each spin-cast BCP film was divided into two pieces. For effective comparison, one piece was retained as a reference, while the other underwent further processing using the C-SVA method. This method involved an extremely shallow aluminum sample chamber. The temperature at the bottom of the chamber was regulated by placing a Peltier element (15.4 V/8.5 A, Stonecold) beneath it, with a PT100 sensor positioned on top. Both the sensor and a 12 V/10 A power supply were connected to a controller (TCM U 10A, Electron Dynamics Ltd., Southampton, Great Britain). The direction of the electric current through this system could be controlled via a PC interface, allowing for heating, cooling, or maintaining any sample placed inside the chamber at any temperature within the −15 °C to 65 °C range, with a 0.01 °C precision that could be adjusted from the temperature controller. Additionally, the sample chamber could be filled with controlled amounts of solvent vapors using a nitrogen-based bubbling system connected to a flow meter. More technical details regarding our experimental setup can be found in either of the following references [52,53,54].

To expose thin BCP films to solvent vapors, the procedure began with heating the sample to 40 °C while introducing a regulated amount of solvent vapors into the chamber. This was followed by a gradual decrease in sample temperature (at a rate of 0.3 °C/s) to 15–23 °C. This step was essential to initiate the condensation of solvent vapors onto each BCP film. The appropriate temperature within the 15–23 °C interval depended on the volatility of the solvent used and was determined by observing the continuous swelling of the film through the interference phenomenon (each film changed color upon condensation of more and more solvent vapors). A film was considered to be swollen enough when its initial thickness increased by about 20 times, generating a polymer concentration in “film solution” of about 5% (note that an interference colors–film thickness calibration was produced with the help of AFM prior to film swelling and used to infer the film thickness; the method of film thickness estimation was described in more detail elsewhere [51]). In this swollen state, BCP molecules were well dissolved within the “film solution”. At this point, we reversed the process by beginning to slightly anneal the film towards 27 °C (this time, at a rate of only 0.01 °C/s) and thus to slowly evaporate solvent vapors. As previously demonstrated in other studies [69,70], the initial gradual annealing of the highly swollen films facilitates the slow evaporation of solvent molecules, and these start to interact and self-assemble into various ordered morphologies. Further annealing of the films to a higher temperature of 40 °C leads to complete evaporation of solvent vapors, returning the films to their original thickness while rearranging their microstructures. Schematics depicting the experimental procedure and expected self-assembly mechanism are shown in Figure 1e. The effectiveness of C-SVA in processing and ordering various polymeric and non-polymeric systems can be observed in other recently published reports [52,53,54,71,72]. It is important to note that in this work, we have not studied the effect of the substrate on the self-assembly process, as we only used silicon wafers for the fabrication of thin BCP films (in this case, the interactions between BCPs and the substrate were considered favorable, as all thin BCP films remained highly stable during the swelling/deswelling procedure, without presenting, for example, any signs of dewetting). Moreover, previous AFM studies conducted on the assembly of conjugated polymers have not revealed any clear structural changes when using two distinct substrates [73].

All AFM measurements were conducted using a system from Molecular Devices and Tools for Nano Technology (NT-MDT) mounted on an Olympus IX71 optical microscope in noncontact mode. This AFM system was purchased as a complete unit from Spectrum Instruments Ltd. (Limerick, Ireland). The AFM measurements were performed in air (at room temperature and 30–60% relative humidity), under vibration protection conditions (the AFM tool is placed on a TS-150 table acquired from The Table Stable Ltd., Mettmenstetten, Switzerland), by utilizing high-resolution Noncontact Golden Silicon probes from NT-MDT (probes displayed a tip radius of curvature smaller than 10 nm and a tip height ranging from 14 to 16 μm). These probes were Au-coated on the detector side cantilever. The latter had a length of 125 ± 5 μm and exhibited a resonance frequency in the range of 87 to 230 kHz. All AFM images (256 × 256 lines) were acquired using a scanning speed of a few μm/s, while the setpoint was continuously adjusted to maintain a very soft tapping regime. Finally, it is important to note that to demonstrate the consistency of the observed film morphology across the entire film surface, the AFM images (often of magnifications ranging between 25 × 25 μm^2^ and 0.25 × 0.25 μm^2^) were measured in at least three different regions on each sample.

## 3. Results and Discussions

**Figure 2:**
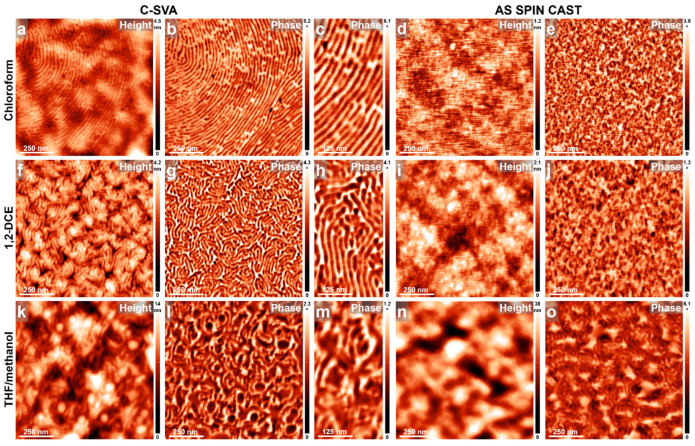
AFM height and phase micrographs emphasizing the morphology of thin films of P4VP_34_-*b*-PB_207_ obtained by spin casting from chloroform (**a**–**e**), 1,2-DCE (**f**–**j**), and THF/methanol (**k**–**o**) solutions, before (**d**,**e**,**i**,**j**,**n**,**o**) and after (**a**–**c**,**f**–**h**,**k**–**m**) their further processing via C-SVA in the corresponding solvent vapors.

In Figure 2, we show the surface of P4VP_34_-*b*-PB_207_ BCP films exposed to various solvent vapors by C-SVA method, alongside their unprocessed spin-cast analogues (for example, a film spin cast from a chloroform solution was exposed to chloroform vapors). AFM results demonstrate that after exposing such a BCP film to chloroform vapors, a smooth, highly ordered structure, represented by periodic parallel stripes with an average lateral periodicity of ~23 nm, was generated (Figure 2a–c). Moreover, parallel stripes consisted of both darker and lighter domains, most probably corresponding to the softer PB and harder P4VP blocks, respectively. This structure formed through the lamellar microphase separation process that arranged the PB blocks between the P4VP domains. In contrast, the unexposed reference film exhibited a surface covered with featureless structures (Figure 2d,e).

Similar results were further obtained by replacing chloroform vapors with 1,2-DCE vapors (Figure 2f–h). Notably, the domains composed of long parallel stripes became smaller, with the stripes exhibiting higher curvatures (compare Figure 2b with Figure 2g). This effect is even more pronounced when replacing once more the 1,2-DCE vapors with more selective solvent vapors coming from a mixture of THF/methanol (Figure 2k–m). In this case, although the lateral dimensions remain similar, the stripes lose their periodicity, resulting in a disrupted and less ordered morphology (obviously, no ordering was observed in either of the unprocessed reference film analogues, as can be seen in Figure 2i,j,n,o). This type of surface morphology can be explained by assuming the quality of the utilized solvent vapors with respect to the employed BCP system, inclusively by considering the Flory–Huggins interaction parameter (χ). The latter measures the interaction of the polymer chains with the solvent molecules, as well as the polymer–polymer interactions (typically, compounds are considered to be miscible when χ is smaller than 0.5 or phase-separated when χ is higher than 0.5) [74]). For example, the low value of χ = 0.022 [75] for the PB–chloroform system indicates chloroform is a good solvent for this block and is capable of promoting chain swelling, favorable interactions, and possibly more extended molecular conformations. Similarly, favorable interactions are also expected for the P4VP–chloroform system (for polyvinylpyridines–chloroform systems, χ was reported to be 0.443 [76]). Additionally, considering the inherently incompatible nature of the P4VP and PB blocks, the efficient phase separation within the P4VP-*b*-PB system in chloroform into highly ordered lamellar structures, as observed in Figure 2a–c, is not surprising in the end. Moreover, 1,2-DCE serves as a good solvent for both the P4VP and PB blocks. While dichloroethane can be regarded as a good solvent for poly(vinylpyridine)s [77,78,79], the χ for PB-1,2-DCE is 0.425 [75], further indicating that 1,2-DCE is also a good solvent for the P4VP-*b*-PB. Consequently, the efficient packing of P4VP_34_-*b*-PB_207_ di-BCP into periodic parallel stripes, as shown in Figure 2f–h, is well justified. Furthermore, while methanol seems a decent/good solvent for poly(vinylpyridine)s (with a corresponding χ = 0.474 [76]), it is a terribly poor solvent for the PB block (χ = 2.694 [75]). Thus, mixing methanol even with a good solvent for PB, such as THF (χ = 0.167 [75]), still generates a poor/selective solvent mixture for P4VP_34_-*b*-PB_207_ BCP. Consequently, the resulting microstructure after C-SVA processing is highly corrupted (Figure 2k–m). Finally, it is important to note that, while similar results obtained for P4VP-*b*-PB di-BCPs in chloroform were previously reported in the literature [52,54], so far, there are no data published on the self-assembly of such di-BCPs in 1,2-DCE and THF/methanol solvents.

**Figure 3:**
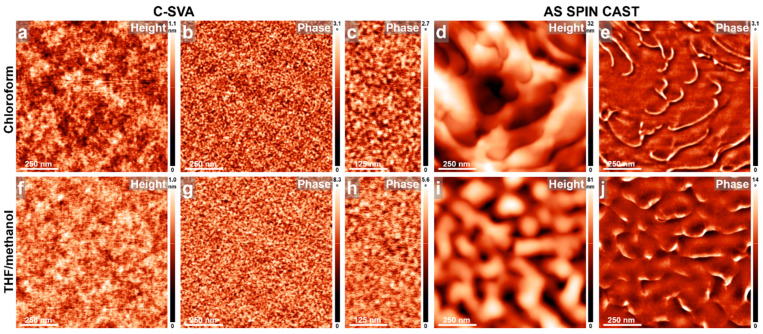
AFM height and phase micrographs emphasizing the morphology of thin films of P4VP_43_-*b*-PB_70_ obtained by spin casting from chloroform (**a**–**e**) and THF/methanol (**f**–**j**) solutions, before (**d**,**e**,**i**,**j**) and after (**a**–**c**,**f**–**h**) their further processing via C-SVA in the corresponding solvent vapors.

When the volume fraction between the two blocks was altered, by increasing the P4VP block from 34 to 43 monomers while simultaneously decreasing the PB block from 207 to only 70 monomers, the resulting P4VP_43_-*b*-PB_70_ di-BCP system produced micellar structures of an average diameter of 16.7 ± 3 nm when exposed to chloroform and THF/methanol vapors. These structures uniformly covered the entire film surface in a smooth manner (roughness was smaller than 1.1 nm, Figure 3a–c,f–h). Considering the interaction parameters χ between chloroform and the two blocks discussed in the previous section, PB–chloroform interactions appear to be the most favorable, likely positioning the PB block on the outer side (corona) of the P4VP_43_-*b*-PB_70_ micelles, while the P4VP block forms the core. Conversely, when exposing P4VP_43_-*b*-PB_70_ di-BCP to a mixture of THF/methanol vapors—where methanol is a very poor solvent for PB—it is possible that the resulting P4VP_43_-*b*-PB_70_ micelles consisted of a P4VP corona and a PB core. In contrast, thin films of P4VP_43_-*b*-PB_70_ that were spin cast from chloroform, but not exposed to solvent vapors, displayed a scaly, layered morphology (Figure 3d,e). A similar scaly morphology, albeit less pronounced, was also observed in reference P4VP_43_-*b*-PB_70_ films spin cast from the THF/methanol mixture (Figure 3i,j). The above experiments conducted on P4VP_43_-*b*-PB_70_ di-BCP could be significant, particularly in the design of efficient antimicrobial surfaces that should exhibit all (quaternized) P4VP blocks at the polymer–air interface.

**Figure 4:**
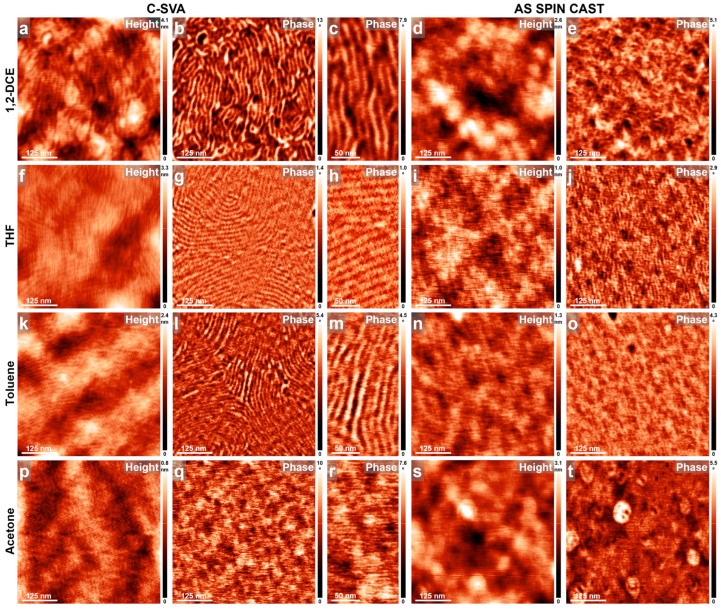
AFM height and phase micrographs emphasizing the morphology of thin films of P2VP_37_-*b*-PB_188_ obtained by spin casting from 1,2-DCE (**a**–**e**), THF (**f**–**j**), toluene (**k**–**o**), and acetone (**p**–**t**) solutions, before (**d**,**e**,**i**,**j**,**n**,**o**,**s**,**t**) and after (**a**–**c**,**f**–**h**,**k**–**m**,**p**–**r**) their further processing via C-SVA in the corresponding solvent vapors.

To investigate the behavior of other poly(vinylpyridine)s alongside PB, the P4VP block was substituted with P2VP, while maintaining a similar number of P2VP and PB monomers to the di-BCP presented in Figure 2 (in this latter case, the 34-monomer-long P4VP block was replaced with a 37-monomer-long P2VP block, while the PB block was shortened by an average of 19 monomers). Periodic parallel stripes were observed after exposing resulting P2VP_37_-*b*-PB_188_ films to vapors of 1,2-DCE, THF, and toluene, respectively (Figure 4a–c, Figure 4f–h, and Figure 4k–m). No such stripes, besides smooth featureless surfaces, were detected in unexposed reference films of P2VP_37_-*b*-PB_188_ that were spin cast from the corresponding solvents (Figure 4d,e,i,j,n,o). Again, the darker domains likely correspond to the softer [80] PB block, while the lighter domains represent the stiffer [81] P2VP block. The average lateral periodicity of P2VP_37_-*b*-PB_188_ di-BCP stripes, inferred by considering many cross-sectional AFM measurements in several samples exposed to the three solvents, was determined to be 13.6 ± 0.5 nm. This value was significantly lower than the 23 nm measured for P4VP_34_-*b*-PB_207_ and included contributions from both the dark-PB and light-P2VP domains, each averaging less than 7 nm in lateral periodicity. This is approximately half of the average periodicity of 13.6 nm observed in the parallel stripes of P2VP_37_-*b*-PB_188_. Note that previous studies have indicated that the P2VP chains were not fully extended within the observed lamellar stripe-like morphology due to unfavorable interactions of P2VP with a poor solvent like toluene [82,83] (the width of ~7 nm observed for the lighter P2VP domains was smaller than the calculated width of over 9 nm expected for the P2VP chains that adopt fully extended conformations [54]). Furthermore, our AFM observations suggest that P2VP_37_-*b*-PB_188_ chains that were exposed to vapors of THF and 1,2-DCE adopt an extended conformation. Despite the fact that the P2VP block is expected to experience unfavorable interactions with a poor solvent like THF, its interactions with the good 1,2-DCE solvent are instead favorable and thus support more extended chain conformations. Nonetheless, fully extended conformations of P2VP chains were not observed experimentally in either of the two cases of self-assembly induced upon C-SVA processing. Finally, no periodic structures/stripes were observed for thin P2VP_37_-*b*-PB_188_ films before and after being exposed to poor solvent vapors of acetone (Figure 4p–t). In conclusion, it appears that when P2VP_37_-*b*-PB_188_ films were exposed via C-SVA to solvent vapors that are good solvents for at least one of the blocks, periodic lamellar structures composed of parallel stripes can be generated; otherwise, as anticipated, such structures do not form.

**Figure 5:**
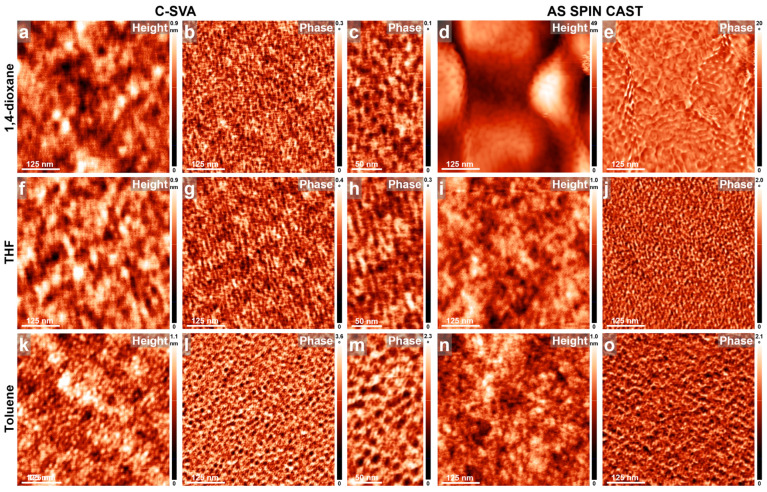
AFM height and phase micrographs depicting the morphology of thin films of P2VP_107_-*b-*P*t*BMA_52_-*b*-PCHMA_604_ obtained by spin casting from 1,4-dioxane (**a**–**e**), THF (**f**–**j**) and toluene (**k**–**o**) solutions, before (**d**,**e**,**i**,**j**,**n**,**o**) and after (**a**–**c**,**f**–**h**,**k**–**m**) their further processing via C-SVA in the corresponding solvent vapors.

To generate additional self-assembled nanostructures by using C-SVA processing, we further exposed micellar tri-BCPs such as P2VP_107_-*b-*P*t*BMA_52_-*b*-PCHMA_604_ to various solvent vapors. Figure 5 compares tri-BCP films exposed via C-SVA to 1,4-dioxane, THF, and toluene vapors to their unexposed reference film analogues. All three tri-BCP films led, upon their C-SVA processing in corresponding solvent vapors, to smooth surfaces (roughness~1 nm) covered with spherical structures, with average diameters (inferred from measuring more than 20 distinct structures) ranging from 16.7 ± 2.5 nm (for the film exposed to THF vapors) to 17.6 ± 2.5 nm (for the film exposed to 1,4-dioxane vapors) to 18 ± 2.5 nm (for the film exposed to toluene vapors). These structures appeared to be further packed either into honeycomb-resembling configurations (as seen in the film exposed to 1,4-dioxane and toluene vapors in Figure 5a–c and Figure 5k–m, respectively) or into parallel stripe-like periodic superstructures (as seen in the film exposed to THF vapors in Figure 5f–h). Given that the P2VP_107_-*b-*P*t*BMA_52_-*b*-PCHMA_604_ tri-BCP system was meticulously designed to form micelles in solution by controlling the volume fraction of the constituent blocks [33], we concluded that the spherical structures described above were indeed micellar objects. Furthermore, based on previous studies [54], the average diameter of P2VP_107_-*b-*P*t*BMA_52_-*b*-PCHMA_604_ micelles formed in films via C-SVA processing is expected to be smaller than the average diameter of analogous micelles in solution due to the contraction of the former during solvent evaporation and subsequent film drying.

In comparison, the reference films of P2VP_107_-*b-*P*t*BMA_52_-*b*-PCHMA_604_ obtained by spin casting from 1,4-dioxane and toluene solutions, and not exposed to solvent vapors, displayed either a significantly rougher surface morphology (with roughness measuring several tens of nanometers) comprised of 200–300 nm porous structures (Figure 5d,e) or a much smoother surface (with roughness of ~1 nm) covered with fewer, rather randomly distributed micellar structures (Figure 5n,o). In contrast, when the tri-BCP film was spin cast from THF, a greater number of micellar structures formed; however, they remained randomly distributed across the surface (Figure 5i,j). Consequently, no packing of micelles into regular honeycomb- or stripe-like configurations could be detected on the surfaces of the unexposed P2VP_107_-*b-*P*t*BMA_52_-*b*-PCHMA_604_ films.

**Figure 6:**
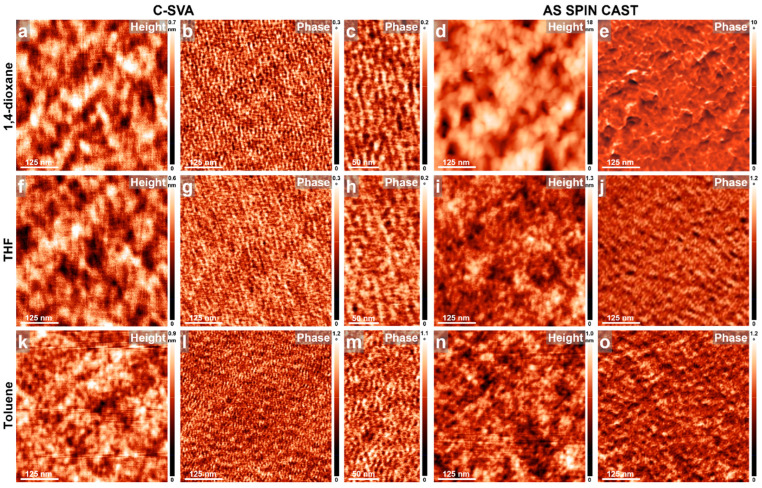
AFM height and phase micrographs depicting the morphology of thin films of P2VP_25_-*b-*P*t*BMA_12_-*b*-PCHMA_173_ obtained by spin casting from 1,4-dioxane (**a**–**e**), THF (**f**–**j**), and toluene (**k**–**o**) solutions, before (**d**,**e**,**i**,**j**,**n**,**o**) and after (**a**–**c**,**f**–**h**,**k**–**m**) their further processing via C-SVA in the corresponding solvent vapors.

Smaller micelles were further obtained by replacing the long P2VP_107_-*b-*P*t*BMA_52_-*b*-PCHMA_604_ with a significantly shorter P2VP_25_-*b*-P*t*BMA_12_-*b*-PCHMA_173_ tri-BCP. The morphologies of films processed via C-SVA in 1,4-dioxane, THF, and toluene vapors are illustrated in Figure 6. The surfaces of the P2VP_25_-*b*-P*t*BMA_12_-*b*-PCHMA_173_ films exposed to 1,4-dioxane and THF vapors were both covered with micellar structures averaging 12.5 ± 1 nm in diameter, as shown in Figure 6a,b,f,g. In contrast, the third film exposed to toluene vapors produced micelles with a significantly smaller average diameter of 8.5 ± 0.7 nm (Figure 6k,l). In all three cases, the micelles were further packed in (rather parallel) stripe-like superstructures, as observed in Figure 6c,h,m. Conversely, micelles obtained from the same tri-BCP in solution were larger, with a reported hydrodynamic radius of 17.9 nm [33]. This discrepancy is attributed to the contraction of the micelles reported here upon the evaporation of solvent vapors, followed by the drying of the film.

Instead, the as spin-cast P2VP_25_-*b*-P*t*BMA_12_-*b*-PCHMA_173_ reference films that were not exposed to solvent vapors exhibited either a predominantly rough “porous” surface morphology when spin cast from 1,4-dioxane solutions (Figure 6d,e) or smooth surfaces covered with fewer, randomly distributed micellar structures when spin cast from THF and toluene solutions (Figure 6i,j,n,o). No further packing of micelles into (parallel) stripe-like superstructures could be clearly observed on the unexposed surfaces of the reference films. Moreover, the average diameter of micelles formed in the P2VP_25_-*b*-P*t*BMA_12_-*b*-PCHMA_173_ film spin cast from toluene was 13.8 ± 0.8 nm, not 8.5 ± 0.7 nm as observed for its analogue processed via C-SVA (compare Figure 6l with Figure 6o). The former value of micellar diameter is higher than the 12.5 ± 1 nm measured for micelles formed in films exposed to 1,4-dioxane and THF vapors. This is not surprising because (i) both 1,4-dioxane and THF are good solvents for all P2VP, P*t*BMA, and PCHMA blocks, while toluene is only a good solvent for the P*t*BMA and PCHMA blocks, being a relatively poor/selective solvent for P2VP (thus, there is good miscibility between P2VP and P*t*BMA in THF, dictated by a lower value of the interaction parameter χ = 0.4 [84]; in contrast, in toluene, these two blocks tend to phase-separate, as χ = 0.7 is higher [84]), and (ii) tri-BCP micelles generally lead to smaller micelles in good/less selective solvents and, thus, to larger micelles in poor/more selective solvents [85]. What remains intriguing is the observed reduction of micellar diameter, from 13.8 ± 0.8 nm to 8.5 ± 0.7 nm, when spin-casting films from toluene solutions and subsequently exposing them via C-SVA to toluene vapors. This clearly demonstrates that C-SVA processing in toluene leads to significant changes in conformational arrangements of P2VP_25_-*b*-P*t*BMA_12_-*b*-PCHMA_173_ macromolecules. To establish the exact nature of these changes, further experimental and structural measurements will be needed in the future. Finally, it is worth mentioning that, while similar micellar structures and their packing into stripe-like superstructures were recently reported for P2VP_25_-*b*-P*t*BMA_12_-*b*-PCHMA_173_ films exposed to 1,4-dioxane vapors [54], no such structures have been reported for films exposed to toluene and THF. Moreover, no micellar structures have been reported so far in films of longer P2VP_107_-*b-*P*t*BMA_52_-*b*-PCHMA_604_ in any of the three solvents.

**Figure 7:**
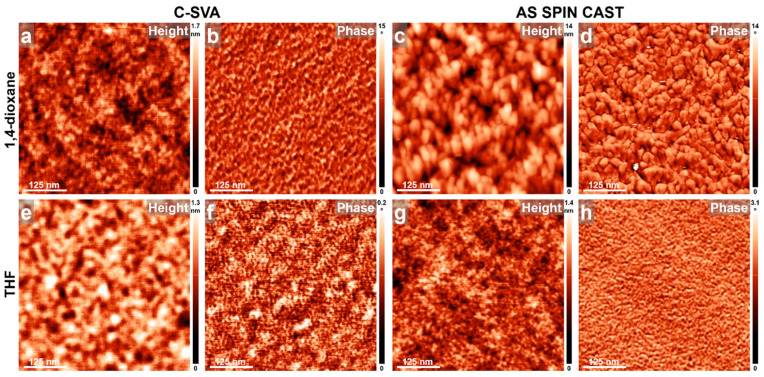
AFM height and phase micrographs showing the morphology of thin films of PAA_31_-*b*-PCHMA_207_-*b-*PAA_31_ obtained by spin casting from 1,4-dioxane (**a**–**d**) and THF (**e**–**h**) solutions, before (**c**,**d**,**g**,**h**) and after (**a**,**b**,**e**,**f**) their further processing via C-SVA in the corresponding solvent vapors.

Because micellar complexes based on PCHMA glassy [86] block could be utilized as gasoline additives or colloidal stabilizers in non-aqueous media [35,87,88], while PAA-*b*-PCHMA experiences adsorption on pigments and, thus, has implications in motor-oil lubricants [35] (for instance, the relationship between self-assembled structures and lubrication behavior was shown to impact lubrication performance [89,90,91,92,93]), it is important to further investigate the generation of micellar structures in the PAA- and PCHMA-based BCPs as well. For that, we spin-cast films of PAA_31_-*b*-PCHMA_207_-*b*-PAA_31_ tri-BCP and exposed them to solvent vapors using the C-SVA method. The obtained results are summarized in Figure 7 and include AFM measurements of the dry films before and after their exposure to 1,4-dioxane and THF vapors. The exposure of films to both solvents led to micellar structures (Figure 7a,b,e,f). However, no clear packing into honeycomb or stripe-like superstructures was observed. The generated micellar structures were randomly distributed across the relatively smooth surfaces. Their average diameters were approximatively 15 ± 1.5 nm in films exposed to 1,4-dioxane vapors and about 14.1 ± 1.5 nm in films exposed to THF vapors. Interestingly, micellar structures of a similar diameter were already present in the tri-BCP film immediately after spin casting from a THF solution (Figure 7g,h). This indicates that C-SVA processing had little to no impact on the self-assembly process of PAA_31_-*b*-PCHMA_207_-*b*-PAA_31_ molecules. In contrast, such structures were absent in a reference film that was spin cast from a 1,4-dioxane solution (Figure 7c,d). In this latter case, the film surface was covered with much larger objects (ranging from a few to several tens of nanometers in lateral dimension) that were less spherical and more irregular in shape. This demonstrates that controlled evaporation of solvent vapors during C-SVA processing is necessary to generate PAA_31_-*b*-PCHMA_207_-*b*-PAA_31_ micellar structures from 1,4-dioxane solutions. Finally, while micelle formation in P2VP-*b*-PCHMA [54] and PCHMA-*b*-PAA [52] di-BCP films has been reported in the literature, no such observations have been made until now for PAA_31_-*b*-PCHMA_207_-*b*-PAA_31_ tri-BCP films.

## 4. Conclusions

By employing an advanced solvent vapor annealing method, we have successfully swollen various BCP thin films in different types of solvent vapors and thus generated (hierarchically) self-assembled surfaces. The latter feature either periodic lamellar structures of a molecular dimension obtained from P4VP_34_-*b*-PB_207_ and P2VP_37_-*b*-PB_188_ di-BCP films exposed to solvents, such as chloroform, 1,2-DCE, THF, and toluene, or simple micellar structures generated from the exposure of films of P4VP_43_-*b*-PB_70_ and PAA_31_-*b*-PCHMA_207_-*b-*PAA_31_ to vapors of chloroform and THF/methanol, on the one hand, and vapors of 1,4-dioxane and THF, respectively. Micellar structures often appeared to be further packed into (parallel) stripe-like superstructures when tri-BCP films made of P2VP_25_-*b-*P*t*BMA_12_-*b*-PCHMA_173_ were exposed to 1,4-dioxane, THF, and toluene. The same was found to be valid for films made of P2VP_107_-*b-*P*t*BMA_52_-*b*-PCHMA_604_ and further exposed to THF vapors. Instead, when P2VP_107_-*b-*P*t*BMA_52_-*b*-PCHMA_604_ films were exposed to 1,4-dioxane and toluene, micellar structures preferred to pack further into honeycomb-resembling configurations. These findings mark progress toward the development of self-assembled nanostructures and surfaces of controlled functionalities, which may ultimately be used in targeted antimicrobial applications (quaternized P2VP and P4VP), as gasoline additives or colloidal stabilizers (PCHMA), in fabrication of motor-oil lubricants (PAA/PCHMA), in advanced manufacturing/coating nanocomposites, etc.

## Data Availability

The original contributions presented in this study are included in the article. Further inquiries can be directed to the corresponding author.
